# Social transition in young trans people accessing gender-affirming care

**DOI:** 10.1080/26895269.2025.2516678

**Published:** 2025-06-20

**Authors:** Pip Buckingham, Michelle A. Tollit, Grace Wong, Rushani Wijesuriya, Monsurul Hoq, Carmen C. Pace, Ken C. Pang

**Affiliations:** a Royal Children’s Hospital, Melbourne, VIC, Australia; b Murdoch Children’s Research Institute, Parkville, VIC, Australia; c Department of Pediatrics, University of Melbourne, Parkville, VIC, Australia

**Keywords:** Adolescents, children, social affirmation, social transition, transgender persons

## Abstract

**Background:**

In-depth quantitative study of how young people undertake social transition is lacking. We aimed to provide a comprehensive profile of social transition among young people accessing gender-affirming care.

**Methods:**

In this cross-sectional study (*n* = 482), young people aged 8–17 years completed online questionnaires before their first appointment at a pediatric gender clinic. Measures captured social transition in different contexts (home, school, online, friends, and extended family) by assessing extent of social transition, and changes to presentation, name and pronouns or intent to make these changes. Young people also reported their concerns about socially transitioning.

**Results:**

The vast majority expressed preferences to socially transition by changing their presentation (96.0%), name (89.6%), and/or pronouns (94.3%). Compared to presentation (74.4%), fewer young people had changed their name (45.8%) or pronouns (44.1%) across all the examined contexts. Social transition was most commonly observed either online or with friends, with home and school being less common contexts, and extended family the least likely context in which to observe change. Overall, there were 129 unique patterns of social transition across contexts, highlighting the heterogeneous nature of social transition. Most young people reported being concerned about socially transitioning (88.8%), with the most prevalent concerns related to bullying, prejudice or discrimination (59.5%).

**Discussion:**

Young trans people show a complex profile of social transition that is context-dependent and likely reflects differences in underlying levels of safety and support. Understanding these nuanced patterns of social transition is necessary to improve clinical care and develop relevant policies and guidelines.

## Introduction

Many trans people socially transition to live authentically in their affirmed gender identities (Ashley, 2019; Coleman et al., 2022). While different definitions exist, social transition is defined in the present study as a component of social affirmation (King & Gamarel, 2021) whereby individuals undertake a process of aligning their gender expression and identity (Coleman et al., 2022; Telfer et al., 2018). Social transition may include practices such as changing one’s presentation (e.g. through clothing, hairstyle, chest binding), name and/or pronouns, and partaking in other gender-aligned activities and behaviors. Because these practices and activities of social transition may be more accessible than affirmation *via* gender-affirming hormones and surgeries, particularly to young trans people (de Vries et al., 2014; Guss et al., 2015)—and because gender is an inherently social phenomenon—social transition is often the first mode of transition these individuals pursue. However, there is no single or correct way to transition or affirm one’s gender. Some trans people may not wish to socially transition at all (Rachlin, 2018), while others aim to reach ‘full transition.’ What constitutes a ‘full transition’ also differs between individuals, as some may not want to undergo social transition in specific ways (Pollitt et al., 2019) or contexts (Olson et al., 2016). Social transition can also be a fluid, ongoing process without a specific end-goal (Durwood et al., 2022; Malatino, 2019), where people can trial different forms of expression to explore and discover their identities (Ashley, 2019).

Although the clothes people wear and the names and pronouns they use are not practices related inherently to health (Gill-Peterson, 2024), the reported positive outcomes of social transition for young trans people in medical research—including improvements in psychosocial functioning compared to pre-transition (Durwood et al., 2024), similar levels of depression and anxiety to their cisgender siblings and peers (Gibson et al., 2021), or feelings of gender euphoria and/or tempered gender dysphoria (Horton, 2023; Pullen Sansfaçon et al., 2020; Turban, 2017)—have seen social transition-related support being provided as a core psychosocial intervention to those presenting to pediatric gender clinics (Campo-Engelstein, 2019; Coleman et al., 2022; Telfer et al. 2018). This medicalization of social transition has, however, contributed to increasing moral debate about trans young people’s social practices. Reflecting their belief that social transition—like gender-affirming medical treatment—is experimental and with a problematic emphasis on the requirement for gold standard evidence (i.e. randomized controlled trials), some have recommended that social transition in childhood or adolescence should be approached with greater caution (Hall et al., 2024). These groups cite not only concerns that social transition may adversely affect a child’s future gender identity development (Cass, 2024) but also recent studies which failed to find an association between social transition and improved mental health (Morandini et al., 2023; Sievert et al., 2021). Proponents of this framework, who also recommend that parents who are considering supporting their child to socially transition should first seek specialist clinical advice (Cass, 2024), have failed to recognize the psychological effects of an increasingly hostile sociopolitical climate on individuals who socially transition, or that adhering to cisnormative gender roles can constitute significant psychosocial distress for young trans people (Mezzalira et al., 2022).

To move toward healthcare for young trans people that is truly gender-affirming, clinicians, researchers and policy makers alike would benefit from having a deep understanding of what social transition looks like in those presenting to specialist gender services. The consistent use of in-depth quantitative measures of social transition, which capture young trans people’s genuine experiences, is necessary to ensure policy and clinical guidelines are informed by robust research and reflect young people’s clinical needs, and to strengthen the provision of person-centered gender-affirming care (Horton, 2024). Previous attempts adopting quantitative approaches have used relatively parsimonious measures to assess social transition, and in defining constructs to measure young people’s social transition, previous research has placed emphasis on the concept of ‘full social transition’ and frequently overlooked the question of how young people socially transition or how they *want to* socially transition.

The binary operationalization of social transition as either ‘full’ or ‘not full’ (or ‘full’/’none’) has also been common practice. Many studies have classified participants as having fully socially transitioned only if they were living consistently as their identified gender (Katz-Wise et al., 2017; Miller & Grollman, 2015; Morandini et al., 2023; Turban et al., 2021). However, trans young people experience transphobic hostility, violence and disaffirmation across various settings including at school, at home, in public, in bathrooms, in healthcare, in politics, and online (Barbee et al., 2022; Chong et al., 2021; Domínguez-Martínez et al., 2023; Durwood et al., 2021; Herrmann et al., 2024; Hill et al., 2021; McGuire et al., 2022). Since trans youth may express their gender only when and where it is safe to do so (Pullen Sansfaçon et al., 2021), we suggest that all the steps they take to affirm their gender should be recognized. Moreover, eliciting young people’s concerns about socially transitioning—which are likely to include worries about bullying, prejudice or discrimination, fears about not being supported or knowing who to trust, and uncertainty regarding how to socially transition—will situate their reports of transition in greater context and lead to a better understanding of these dynamics.

Others have operationalized social transition as a tripartite construct (i.e. no, partial or full transition) using a variety of approaches. Steensma et al. (2013), for example, assessed social transition *practices*, and defined partial transition as having changed one’s clothing and hairstyle but not name or pronouns, and full transition as having undergone all these changes. As young people face systemic, administrative and interpersonal constraints to using pronouns and chosen name in settings such as school or healthcare institutions (Belmont et al., 2024; DeChants et al., 2022; Pollitt et al., 2019), it may be reasonable to assume that name and pronoun changes signify a greater extent of social transition than changes in presentation alone. However, without ascertaining whether young people want to engage in these social transition practices, this researcher-prescribed categorization potentially overemphasizes the weight of specific social practices and gives rise to a monolithic portrayal of what social transition comprises. As an alternative, directly asking young people to state how much they’ve socially transitioned on a quantitative scale could better depict their *extent* of social transition in accordance with their own goals. Other studies have operationalized social transition as a tripartite construct based on the *contexts* (e.g. home, school, and online) in which social transition has occurred. For example, both Sievert et al. (2021) and Levitan et al (2019) defined partial social transition as living as one’s affirmed gender in some but not all contexts and full social transition as doing so in all contexts. Examining how social transition occurs *within* different contexts may further deepen our understanding of this process across various environments young people navigate.

Importantly, most of the above approaches to quantifying social transition presume young people implement a prescriptive, linear trajectory or assemblage of social practices. This is consistent with the well-documented history of health professionals gatekeeping gender-affirming care from trans people who haven’t transitioned linearly across gender binaries (Johnson, 2016) or achieved full social transition (Chong et al., 2021; Horton, 2022; Kearns et al., 2021). Such practices reflect the medical establishment’s historically narrow acceptance and reproduction of transnormativity and transmedicalization, contributing to institutional violence against those with divergent identities and embodiment goals.

Moving forward, it will be crucial to find better ways to describe and understand the diverse ways young people socially transition. The overarching aim of this study was therefore to comprehensively describe the nature of social transition among children and adolescents aged 8–17 years presenting for the first time to a specialist pediatric gender service in Victoria, Australia. To address this aim, we specifically sought to (i) describe the young person’s self-reported extent of social transition and how this differed between contexts (home, school, online, with friends, and with extended family); (ii) describe the social transition practices (name, appearance and pronouns) most preferred and used overall and in different contexts; (iii) summarize young people’s most pertinent concerns about social transition; and (iv) describe how the overall extent of social transition differed between those with and without concerns.

## Methods

### Design, setting and participants

Cross-sectional data were drawn from the Royal Children’s Hospital Gender Service (RCHGS), which provides state-wide multidisciplinary gender-affirming care for young people living in Victoria, Australia. As part of routine clinical care, young people and their parents/caregivers complete surveys at regular intervals to monitor the young person’s health and wellbeing over time. A total of 618 young people who attended their first RCHGS appointment between February 2017 and February 2020 were recruited to the Trans20 cohort (Tollit et al., 2019), which provides the basis for the current investigation and has been approved by the Royal Children’s Hospital Human Research Ethics Committee (#36323). Explicit consent to report on these clinical audit data was not deemed necessary by the approving ethics committee.

Separate baseline questionnaires were completed by young people and a parent/caregiver approximately 0–4 wk before their first appointment. At this time, they were advised that their responses would not influence their access to care, and that parents of younger patients could assist their child in completing the survey. Young people indicating *via* two questions that they had socially transitioned in some way or intend to in the future were included in the final analytic sample (see Figure 1).

**Figure 1.**
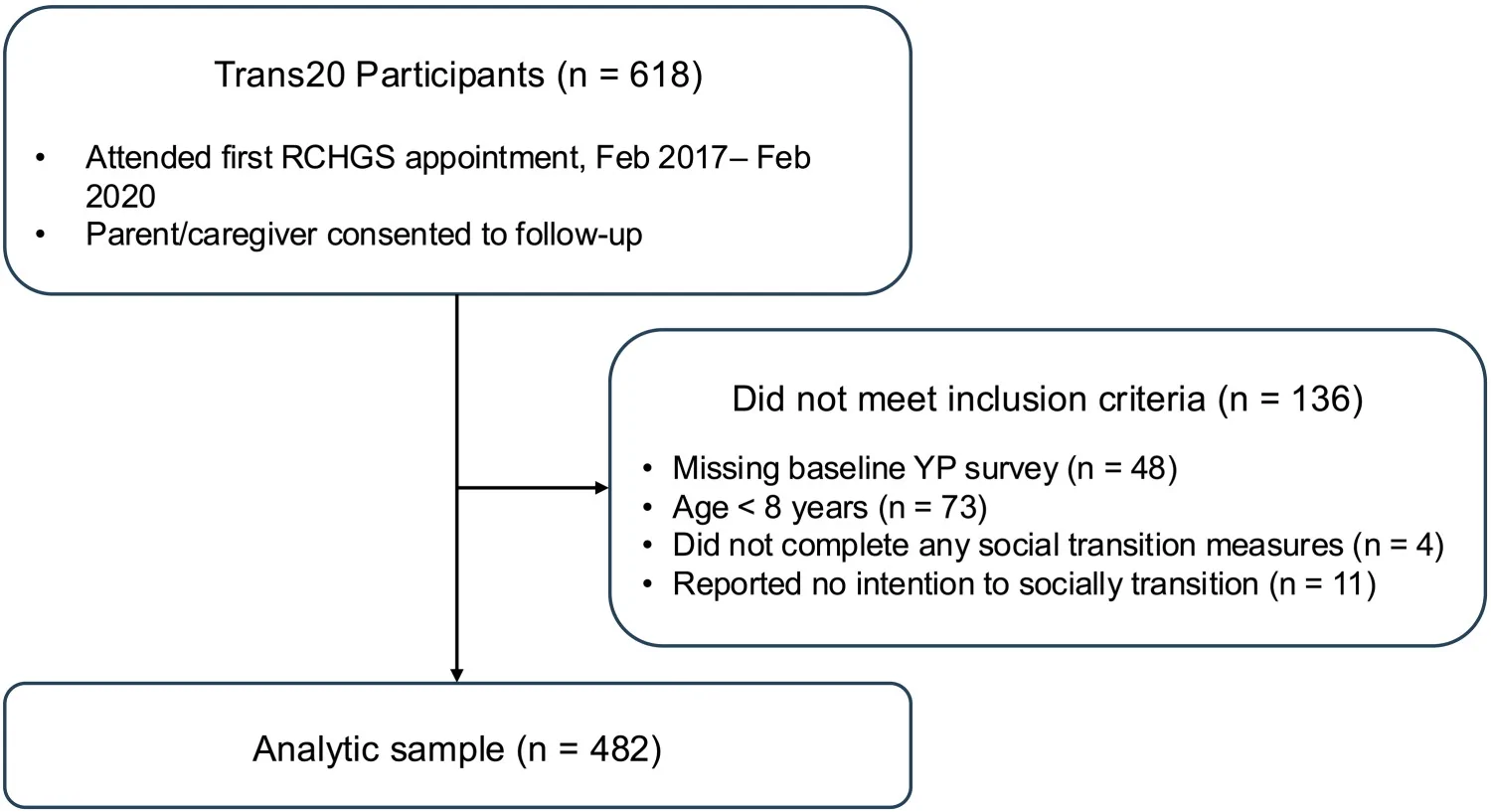
Participant flow chart for the analytic sample. A total of 570 out of 618 Trans20 participants (92.2%) completed a baseline survey prior to their first Royal Children’s Hospital Gender Service (RCHGS) appointment. To be included in the analytic sample, young people (YP) needed have indicated that they had socially transitioned in some way (*n* = 399) or might want to do so in the future (*n* = 83) *via* two questions in the surveys (see Supplementary Files: Table S1), and be 8–17 years old at the time of their first appointment (social transition measures are not included in surveys for those <8 years). Of the 497 individuals aged 8–17 who completed the baseline survey, 96.9% (*n* = 482) were included in the sample. Of the 15 who were excluded, 11 responded ‘no’ to both questions and four did not provide a response.

Sample demographics such as gender, age, country of birth, language spoken at home, Aboriginal and/or Torres Strait Islander descent and education setting were collected using young person and parent surveys and the patient Electronic Medical Record (EMR). Socioeconomic status and rurality were determined *via* linkage of young people’s residential postcodes with Index of Relative Socioeconomic Advantage and Disadvantage (IRSAD) (Australian Bureau of Statistics, 2021) and Modified Monash Model (Australian Government Department of Health and Aged Care, 2024), respectively.

#### Social transition constructs and measures

Below we outline the social transition *constructs* of interest, and the relevant measures used for measuring each. Detailed definitions and the derivation of the measures are provided in Table 1. The questionnaire is provided in Supplementary Table S1.

**Table 1.** Constructs, variables and coding rules.

Construct		Variables	Data	Values	Derivation
Social transition extent					
1	Social transition extent	Separate variable for each of the contexts: Home School Online (YP aged ≥12) Friends Extended Family	Ordinal	1 ‘In no ways’ 2 ‘In some ways’ 3 ‘In many ways’ 4 ‘As much as I can’ 5 ‘Not applicable’	N/A.
2	Any extent of social transition	Separate variable for each of the contexts: Home School Online (YP aged ≥12) Friends Extended Family	Binary	0 ‘No’ 1 ‘Yes’	Derived from corresponding #1 variable: 0 ‘No’ if responded ‘In no ways’ 1 ‘Yes’ if responded ‘In some ways’, ‘In many ways’ or ‘As much as I can’
3	Overall extent of social transition	Single variable	Continuous	Scale ranging from 1 to 4 with 1 referring to having socially transitioned ‘In no ways’ in every context and 4 referring to having socially transitioned ‘As much as I can’ in every context.	Sum of the values of the ordinal social transition extent variables (#1) divided by number of contexts (5 contexts for YP aged ≥12 and 4 contexts for those <12 years). Scale omits individuals who selected ‘not applicable’ or did not provide a response to ≥ 1 context.
Social transition practices and preferences					
4	Change of presentation	Separate variable for each of the contexts: Home School Online (YP aged ≥12) Friends Extended Family	Nominal	1 ‘Done’ 2 ‘One day’ 3 ‘I don’t want to’ 4 ‘I don’t know’ 5 ‘Not applicable’	N/A
5	Change of name	Separate variable for each of the contexts: Home School Online (YP aged >12) Friends Extended Family	Nominal	1 ‘Done’ 2 ‘One day’ 3 ‘I don’t want to’ 4 ‘I don’t know’ 5 ‘Not applicable’	N/A
6	Change of pronouns	Separate variable for each of the contexts: Home School Online (YP aged >12) Friends Extended Family	Nominal	1 ‘Done’ 2 ‘One day’ 3 ‘I don’t want to’ 4 ‘I don’t know’ 5 ‘Not applicable’	N/A
7	Preference to change presentation (proximal measure)	Separate variable for each of the contexts: Home School Online (YP aged >12) Friends Extended Family	Nominal	0 ‘No’ 1 ‘Yes’ 2 ‘Don’t know’	Derived from corresponding #4 variable: 0 ‘No’ if responded ‘I don’t want to’ 1 ‘Yes’ if responded ‘Done’ or ‘one day’ 2 ‘Don’t know’ if responded ‘I don’t know’
8	Preference to change name (proximal measure)	Separate variable for each of the contexts: Home School Online (YP aged >12) Friends Extended Family	Nominal	0 ‘No’ 1 ‘Yes’ 2 ‘Don’t know’	Derived from corresponding #5 variable: 0 ‘No’ if responded ‘I don’t want to’ 1 ‘Yes’ if responded ‘Done’ or ‘one day’ 2 ‘Don’t know’ if responded ‘I don’t know’
9	Preference to change pronouns (proximal measure)	Separate variable for each of the contexts: Home School Online (YP aged >12) Friends Extended Family	Nominal	0 ‘No’ 1 ‘Yes’ 2 ‘Don’t know’	Derived from corresponding #6 variable: 0 ‘No’ if responded ‘I don’t want to’ 1 ‘Yes’ if responded ‘Done’ or ‘one day’ 2 ‘Don’t know’ if responded ‘I don’t know’
10	Changed presentation	Separate variable for each of the contexts: Home School Online (YP aged >12) Friends Extended Family	Binary	0 ‘No’ 1 ‘Yes’	Derived from corresponding #4 variable: 0 ‘No’ if responded ‘One day’ 1 ‘Yes’ if responded ‘Done’
11	Changed name	Separate variable for each of the contexts: Home School Online (YP aged >12) Friends Extended Family	Binary	0 ‘No’ 1 ‘Yes’	Derived from corresponding #5 variable: 0 ‘No’ if responded ‘One day’ 1 ‘Yes’ if responded ‘Done’
12	Changed pronouns	Separate variable for each of the contexts: Home School Online (YP aged >12) Friends Extended Family	Binary	0 ‘No’ 1 ‘Yes’	Derived from corresponding #6 variable: 0 ‘No’ if responded ‘One day’ 1 ‘Yes’ if responded ‘Done’
13	Overall preference to change [name/ appearance/pronouns] (preference in at least one context)	Separate variables for each social transition practice: Presentation Name Pronouns	Nominal	0 ‘No’ 1 ‘Yes’ 2 ‘Don’t know’	Derived from #4 (presentation), #5 (name), #6 (pronouns), all contexts: 0 ‘No’ if responded ‘No’ in every context; 1 ‘Yes’ if responded ‘done’ or ‘one day’ in ≥1 context; 2 ‘Don’t know’ if responded either ‘I don’t know’ or ‘No’ in every context.
14	Implementation of social transition practices in ≥1 preferred context	Separate variables for each social transition practice: Presentation Name Pronouns	Binary	0 ‘No’ 1 ‘Yes’	Derived from #4 (presentation), #5 (name), #6 (pronouns), all contexts: 0 ‘No’ if responded ‘One day’ in every context; 1 ‘Yes’ if responded ‘Done’ in at least one context.
15	Implementation of social transition practices in all preferred contexts	Separate variables for each social transition practice: Presentation Name Pronouns	Binary	0 ‘No’ 1 ‘Yes’	Derived from #4 (presentation), #5 (name), #6 (pronouns), all contexts: 0 ‘No’ if responded ‘One day’ in ≥1 context; 1 ‘Yes’ if responded ‘Done’ in every context.
Worries about social transition					
16	Any worries about social transition	Single variable: YP is worried about at least one of the following: Safety from bullying, prejudice or discrimination; Knowing who to trust; Support from parents/ caregivers; Support from siblings; Support from school teachers; Support from friends Knowing ways to socially transition; Something else	Nominal	0 ‘No’ 1 ‘Yes’ 2 ‘Don’t know’ (not reported) 3 ‘Prefer not to answer’ (not reported)	Derived from individual items corresponding to each worry or alternative response (e.g., ‘Prefer not to answer’): 0 ‘No’ if responded ‘I am not worried’; 1 ‘Yes’ if selected any worry from the list; 3 ‘Don’t know’ if responded ‘I don’t know’; 4 ‘Prefer not to answer’ if responded ‘prefer not to answer’ Note: Qualitative responses to ‘Something else’ (other worries) were manually checked: three individuals who selected ‘other worry’ were recoded as ‘No’ because they described not being worried in a corresponding free-text response and did not select a different item from the list.
17	Three things that worry you most about socially transitioning	Variable for each ‘worry’: Safety from bullying, prejudice or discrimination; Knowing who to trust; Support from parents/ caregivers; Support from siblings; Support from school teachers; Support from friends Knowing ways to socially transition; Something else (YP reported [item] as one of the top three things that worries them most about socially transitioning).	Binary	0 ‘No’ 1 ‘Yes’	Derived from individual items corresponding to each worry or alternative response (e.g., prefer not to answer): 0 ‘No’ if the item (worry) was not selected but any other response was selected (except for ‘prefer not to answer’ which was coded as missing). 1 ‘Yes’ if the item was selected. Note: Qualitative responses to ‘Something else’ (other worries) were manually checked: four individuals were subsequently coded as ‘No’ in this variable because the YP either described not being concerned in free-text (*n* = 3), or described concerns that duplicated a preexisting multiple choice option (e.g., support from siblings; *n* = 1).

YP = young person.

#### Social transition contexts

The contexts examined in this study were: home, school, with friends, with extended family and online (the latter assessed only in those aged ≥12 years, corresponding to the age that children were legally allowed to access social media platforms in Australia when Trans20 was established). For each context, young people completed questions about the extent of their social transition and their social transition practices (see Supplementary Table S1). Of note, the questions on social transition extent and the contexts ‘friends’ and ‘extended family’ were only incorporated into surveys on 2nd August 2018.

#### Social transition extent

Young people were asked how much they have socially transitioned in each context indicating the extent as: ‘not at all’, ‘in some ways’, ‘in many ways’, ‘as much as I can,’ or ‘not applicable’. We used this to derive the following measures of social transition extent within and across contexts:

1. Ordinal social transition extent within each context (1: ‘In no ways,’ 2: ‘In some ways,’ 3: ‘In many ways,’ 4: ‘As much as I can,’),
2. Any social transition (extent) within each context (Yes, no),
3. Average extent of social transition across all contexts (ranging from 1 to 4, with 1 indicating having socially transitioned ‘In no ways’ in every context and 4 referring to having socially transitioned ‘As much as I can’ in every context).

#### Social transition practices and preferences

Several measures were used to describe social transition practices within each context:

1. Preference for each practice within each context (Yes, No, I don’t know),
2. Implementation of preferred practices within each context (among those who were identified as holding a preference for the practice: Yes, No).
  To identify the most frequently preferred and used social transition practices across contexts we used the following measures:
3. Overall preference for social transition practices across contexts (Yes in at least one context, No in every context, Don’t know in every context),
4. Implementation of social transition practices in more than one preferred context (Among those who were identified as holding a preference for the practice in at least one context: Yes in at least one preferred context, No in every preferred context),
5. Implementation of social transition practices in all preferred contexts (Yes in every preferred context, No in at least one preferred context).

#### Worries about socially transitioning

Worries regarding social transition were recorded by asking young people to select up to three options from a list. The constructs and measures used to assess worries about social transition were:

1. Any worries about social transition (Yes if any worry was selected, No if not worried),
2. Binary variables corresponding to each worry (Yes if selected, No if not selected), to determine the most prevalent of these worries.

### Statistical analysis

Demographic variables of the analytic sample are presented as frequency (*n*), proportion (%) for categorical variables, and mean (*m*) and standard deviation (SD) for continuous variables. As mentioned above, some changes to the social transition measures were implemented after data collection started. The sample was therefore grouped based on survey submission date, and we inspected proportions and 95% CIs to identify potential demographic differences between young people who completed the survey pre/post 2nd August 2018.

Self-reported extent of social transition within each context, social transition practices (preferred or changed), and concerns regarding transition, are presented as the estimated proportion of participants belonging to each category and 95% confidence intervals (CIs). We also estimated the proportions and 95% CIs to answer the first two research questions (i.e. to describe differences in extent of social transition between contexts and which social transition practices are most preferred and used across contexts).

A heatmap (social transition practices preferred and used in different contexts) was generated to visualize any differences between proportions of young people belonging to each category. A further heatmap was generated to observe individual-level patterns of social transition practices among the 208 individuals who provided complete data on social transition extent after 2nd August 2018. To generate this second heatmap, we aggregated those with similar patterns of social transition in Excel by sorting individuals according to the number of contexts in which they reported ‘Done’, ‘One day’ and ‘Don’t know’/’Don’t want to’ respectively regarding changes to presentation, name and pronouns. We then identified and counted unique patterns and described these patterns qualitatively.

To describe how the overall extent of social transition differed according to young people’s concerns about social transition, we estimated the differences in average extent by each of these concerns and any concerns. For this we used a simple linear regression model whereby in separate analyses, the variables for each of the worries (see Table 1) or the variable ‘any worries’ were regressed on the overall extent of social transition. For analyses involving the individual ‘worry’ items, comparisons are reported as the mean difference (95% CI) in extent of social transition between those who indicated [item] was one of the three options that worried them most about socially transitioning and those who did not select this item. The analysis involving the ‘any worries’ variable is reported as the mean difference in extent of social transition between young people who reported any worries about socially transitioning and those who reported not being worried about socially transitioning.

Stata SE/18 (StataCorp, 2023) was used for all analyses unless otherwise specified.

## Results

### Demographic characteristics

Young people’s demographic characteristics at the time of their first RCHGS appointment are shown in Table 2. Of 482 young people, all but four individuals identified with a gender differing from that presumed for them at birth, with more than half the sample identifying as boys (53.3%). The overall sample was 14.5 years old on average (SD = 2.2). Overall, the majority of young people in our sample were born in Australia (92.3%), spoke English as their main language (96.5%), were non-Aboriginal and/or Torres Strait Islander (93.2%), and from more socioeconomically advantaged backgrounds (i.e. IRSAD quintile 5: 30.7%). Demographic characteristics were largely similar between young people who submitted their survey before August 2nd 2018 and those who submitted on/after this date (Supplementary Table S2).

**Table 2.** Patient baseline characteristics (*n* = 482).

	*n* (%)
Gender*	
Boy	257 (53.3)
Girl	87 (18.0)
Nonbinary	78 (16.2)
Unsure	56 (11.6)
Prefer not to answer	2 (0.4)
Missing	2 (0.4)
Age	
8–11 years	75 (15.6)
12–17 years	407 (84.4)
Missing	0 (0.0)
Mean (SD)	14.5 (2.2)
Country of origin	
Australia	445 (92.3)
Other	33 (6.8)
Missing	4 (0.8)
Main language spoken at home	
English	465 (96.5)
Other	5 (1.0)
Missing	12 (2.5)
Aboriginal and/or Torres Strait Islander	
Yes	12 (2.5)
No	449 (93.2)
Missing	21 (4.4)
Attends school	
Yes	442 (91.2)
No	27 (5.6)
Missing	13 (2.7)
School type	
Government (public)	293 (64.4)
Independent (private)	96 (21.1)
Other	34 (7.5)
Missing	32 (7.0)
Socioeconomic status (IRSAD quintile)	
1	45 (9.3)
2	61 (12.7)
3	90 (18.7)
4	137 (28.4)
5	148 (30.7)
Missing	1 (0.2)
Residential location	
Metropolitan	330 (68.5)
Regional center	60 (12.4)
Large rural town	9 (1.9)
Med rural town	16 (3.3)
Small rural town	65 (13.5)
Missing	2 (0.4)

IRSAD = Index of Relative Socioeconomic Advantage and Disadvantage (based on residential postcode) where lower scores indicate higher relative socioeconomic disadvantage.

*To ascertain gender, young people were asked to select all applicable gender identity labels from a list of 12 options, which were later collapsed into five discrete groups (boy/man, girl/woman, nonbinary, unsure, and prefer not to say) as described previously (Blacklock et al., 2021).

### Social transition extent across different contexts

Figure 2 and Supplementary Table S3 show the different social transition extents across different contexts. Many young people had transitioned at least to some extent in all contexts: 85.5% (*n* = 200) at home, 82.7% (*n* = 187) at school, 86.6% (*n* = 161) online, 86.7% (*n* = 202) with friends, and comparatively less (72.8%, *n* = 163) had undergone social transition to some extent with extended family (i.e. 27.2% [*n* = 61] of young people had in ‘no ways’ socially transitioned in this context).

**Figure 2.**
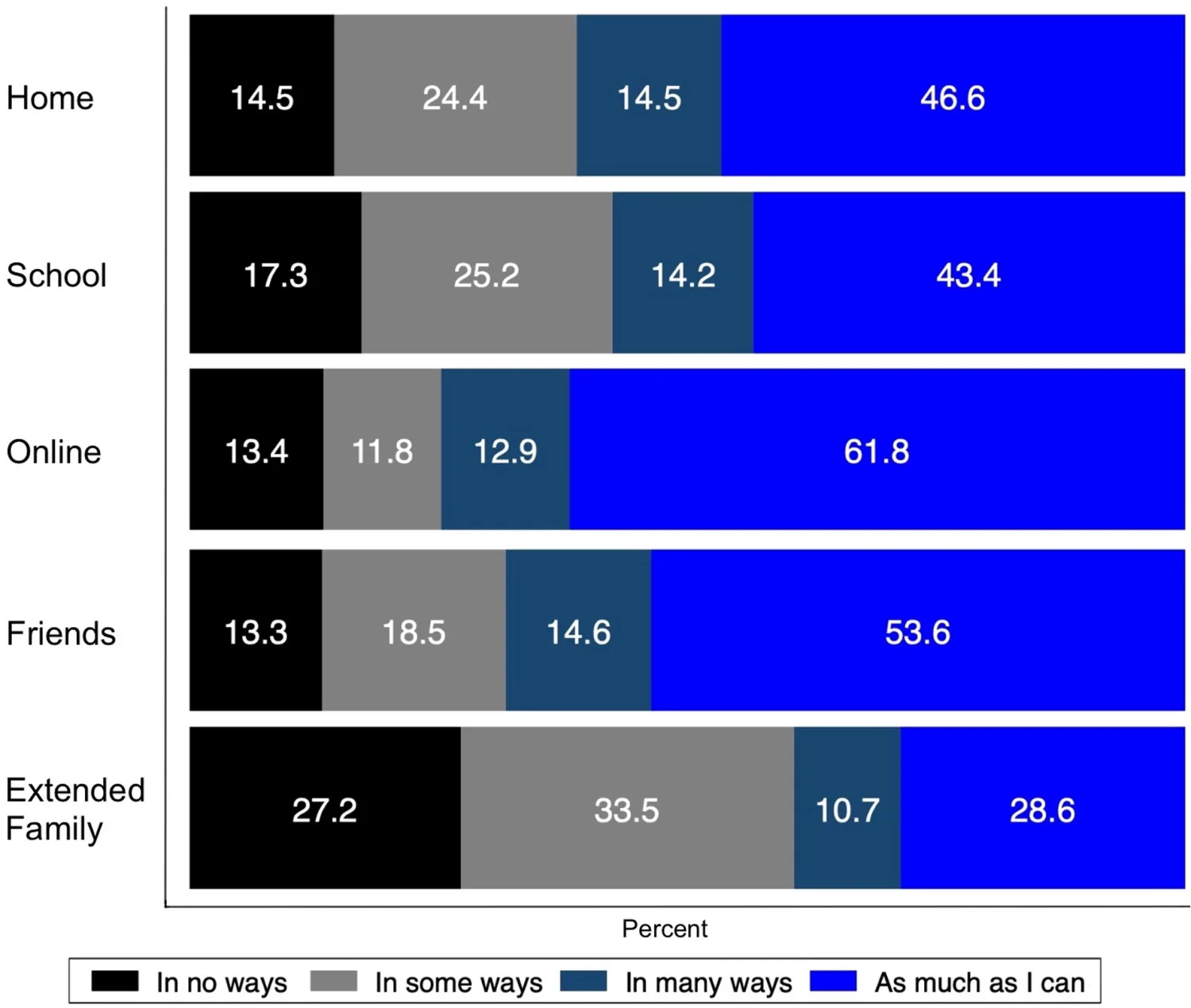
Young people’s reported extent of social transition at home (*n* = 234), at school (*n* = 226), online (*n* = 186), with friends (*n* = 233), and with extend family (*n* = 224).

When specifically looking at ‘as much as possible’ responses, extent of social transition was greatest online (61.8%, *n* = 115) and with friends (53.7%, *n* = 125).

### Social transition practices and preferences

As shown in Table 3, the vast majority of young people had a preference to change their presentation (96.0%, *n* = 457), pronouns (94.3%, *n* = 449) and/or name (89.6%, *n* = 421) to match their gender identity. A small minority of young people did not wish to change either their presentation, name, or pronouns in any context, but this proportion was higher for name (5.3%, *n* = 24) compared other practices [presentation (1.9%, *n* = 9), pronouns (0.6%, *n* = 3)].

**Table 3.** Preference and implementation of social transition practices (presentation, name and pronouns) across contexts (home, school, online, with friends and with extended family).

Change	Presentation (*n* = 476)		Name (*n* = 470)		Pronouns (*n* = 476)	
	n	% (95% CI)	n	% (95% CI)	*n*	% (95% CI)
Preference						
Yes (≥1 context)	457	96.0 (93.8–97.4)	421	89.6 (86.5–92.0)	449	94.3 (91.8–96.1)
No (all contexts)	9	1.9 (1.0–3.6)	25	5.3 (3.6–7.8)	3	0.6 (0.2–1.9)
Don’t know (≥1 context)	10	2.1 (1.1–3.9)	24	5.1 (3.4–7.5)	24	5.0 (3.4–7.4)
Done, ≥1 context						
Yes	397	86.9 (83.4–89.7)	356	84.6 (80.8–87.7)	387	86.2 (82.7–89.1)
No, but will in the future	60	13.1 (10.3–16.6)	65	15.4 (12.3–19.2)	62	13.8 (10.9–17.3)
Done, all contexts						
Yes	340	74.4 (70.2–78.2)	193	45.8 (41.1–50.6)	198	44.1 (39.6–48.7)
No, but will in the future	117	25.6 (21.8–29.8)	228	54.2 (49.4–58.9)	251	55.9 (51.3–60.4)

Preference defined by proxy as having implemented the practice (‘Done’) or intending to in the future (‘One day’).

From Table 3, it was also evident that most of those reporting preference to change their presentation had achieved this to some extent: 86.9% (*n* = 397) had done so in at least one context and 74.4% (*n* = 340) had done so in all contexts they wanted to. Conversely, while the majority had changed their pronouns (86.2%, *n* = 387) or name (84.6%, *n* = 356) in at least one context, just less than half had done so in every context they intended to [pronouns (44.1%, *n* = 198), name (45.8%, *n* = 193)].

Figure 3 (and Supplementary Table S4) displays young people’s patterns of preferring and implementing these social transition practices across each of the five different contexts we examined. While preference to change presentation, name and/or pronouns was generally widespread across contexts, the percentages of young people with preference to change name and pronouns appeared to be lower with extended family than other contexts. It was also evident that those with a preference to change their presentation/name/pronouns in a given context had most frequently made these changes online and with friends. Lower proportions had made these changes at home, and the lowest proportions had made these changes with extended family or at school.

**Figure 3.**
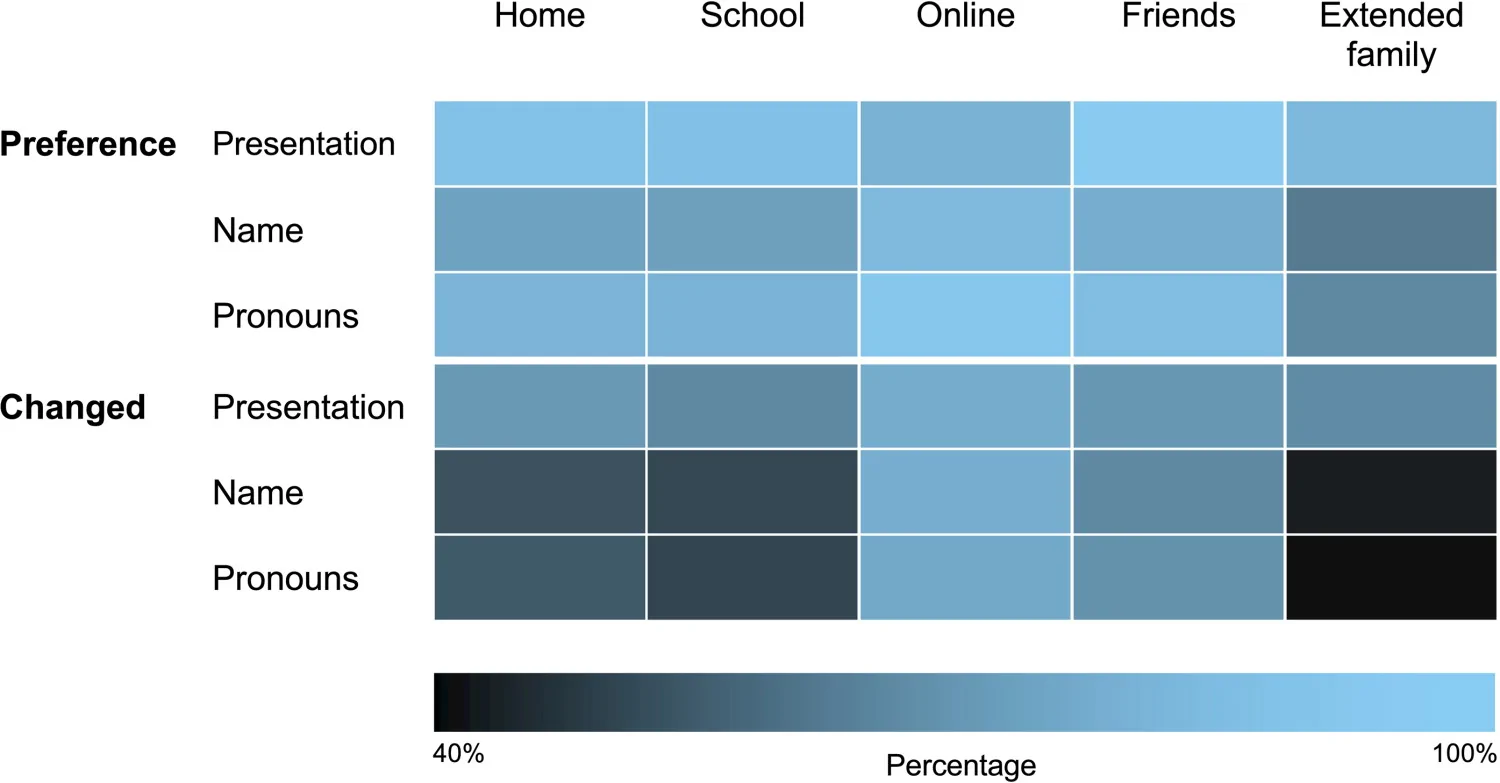
Heatmap showing group-level patterns of preferring and implementing social transition practices across each of the contexts examined. The rows of the heatmap correspond to social transition practices (presentation, name or pronouns) and columns correspond to the contexts examined (home, school, friends, extended family and online – the latter of which was only in those aged 12 or above). In the first three rows, each cell is colored to represent the percentage of young people that indicated a preference to implement the social transition practice in that context. In the latter three rows, each cell is colored to represent the percentage of those who reported having implemented the social transition practice in that context (only among those who held these preferences). These patterns of social transition show that presentation changes occur similarly across all preferred contexts, while young people more frequently change their name and pronouns online and with friends compared to other contexts (proportions and accompanying 95% CIs are provided in Supplementary Table S4).

### Patterns of social transition

These contextual patterns of social transition are also visible in Figure 4, which shows profiles of social transition at an individual level. This figure additionally demonstrates the heterogeneity and complexity of social transition across the entire cohort.

**Figure 4.**
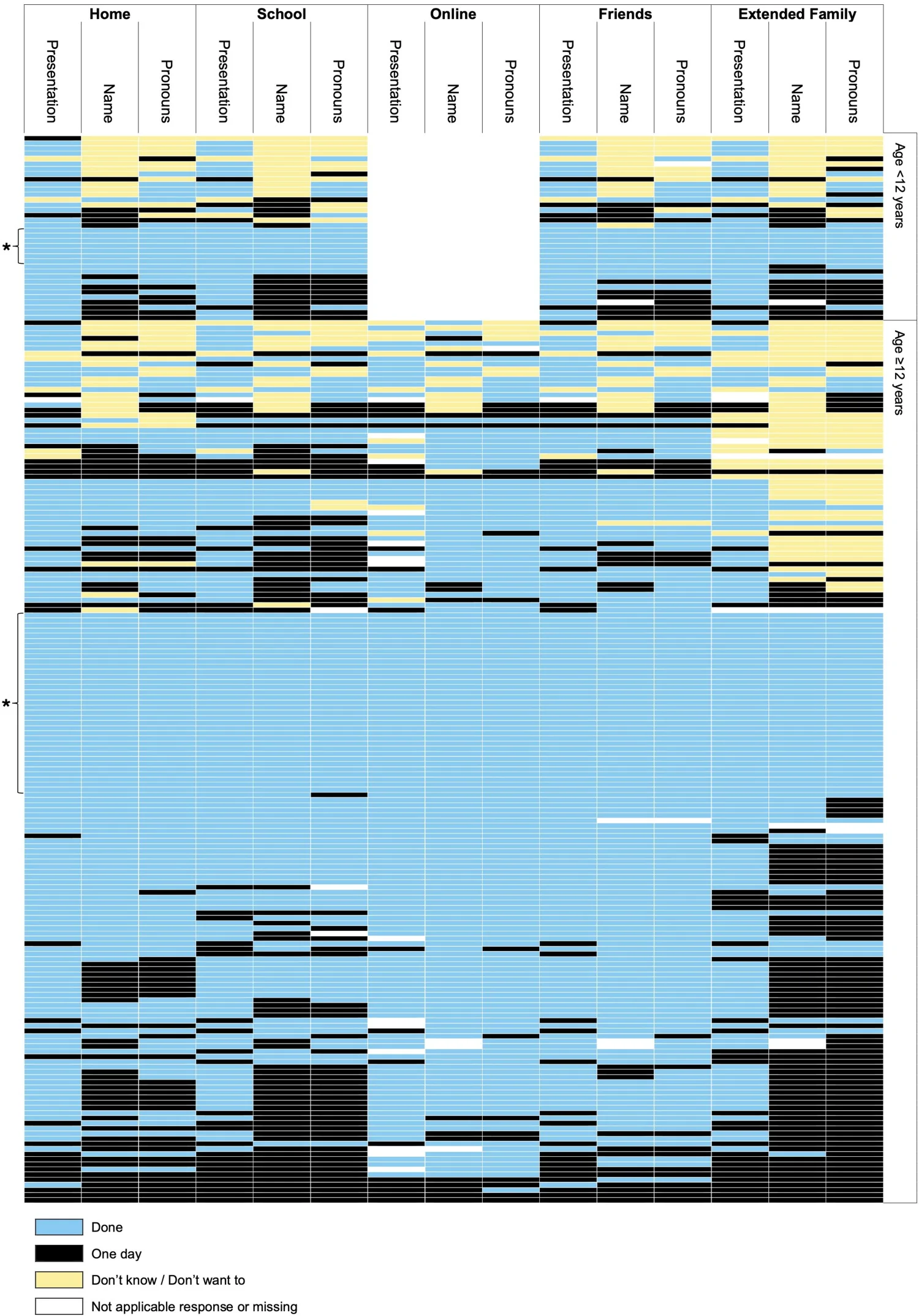
Heatmap showing each young person’s social transition practices across the contexts examined. Each of the 208 individuals for whom we had complete data on social transition extent for all contexts is represented by a single row of the heatmap. Each column corresponds to a particular social transition practice (i.e. presentation, name and pronouns) and context (school, home, friends, extended family or online – the latter of which is only those aged 12 or above), giving 15 columns in total. Each cell is colored either: blue to represent the young person having changed their presentation/name/pronouns in that context; black to represent the young person not having made the change but planning to do so in the future; yellow to indicate a preference not to implement the social transition practice or uncertainty about doing so; and white to indicate not applicable responses (*n* = 1 to *n* = 14) or missing data (*n* = 0 to *n* = 1). Altogether, 129 different patterns of social transition were observed. The most common of these (denoted by *) were shared by 42 young people [≥12 years (*n* = 35), <12 years (*n* = 7)] who had changed their presentation, name and pronouns in all the examined contexts.

In total, there were 129 unique patterns of social transition reported among 208 young people. 110 of these patterns corresponded to a single person in each case. Conversely, the most commonly observed of these patterns were shared by 42 young people [≥12 years (*n* = 35), <12 years (*n* = 7)] who had changed their presentation, name and pronouns in all the examined contexts. Similarly, another 9 individuals [≥12 years (*n* = 8), <12 years (*n* = 1)] had changed their presentation in all examined contexts and had also changed their name and pronouns in all contexts with the exception of extended family. At the other end of the spectrum, ten individuals [≥12 years (*n* = 8), <12 years (*n* = 2)] had yet to make any of their desired changes.

Meanwhile, other patterns highlighted young people’s differences in their intention to socially transition. For example, four individuals [≥12 years (*n* = 1), <12 years (*n* = 3)] had changed their presentation in all contexts but did not intend to change name and pronouns in any context, while two others (both ≥12 years) had changed their presentation and name in all contexts but did not intend to change pronouns, and another four [≥12 years (*n* = 2), <12 years (*n* = 2)] had changed presentation and pronouns in all contexts did not intend to change their name.

### Worries about social transition

As shown in Table 4, the majority of young people reported being worried about socially transitioning (88.8%, *n* = 371). Being safe from bullying, prejudice or discrimination was the most frequently reported concern (59.5%, *n* = 280), followed by not knowing who to trust (44.8%, *n* = 211). Worries that were less frequently reported included not having support from parents/caregivers (21.2%, *n* = 100), siblings (14.0%, *n* = 66), teachers (20.4%, *n* = 96) or friends (22.5%, *n* = 106) or not knowing ways to socially transition (20.2%, *n* = 95).

**Table 4.** Concerns about social transition (*n* = 471).

	Yes		No	
	*n*	% (95% CI)	*n*	% (95% CI)
Any worries	379	88.8 (85.4–91.4)	48	11.2 (8.6–14.6)
Three things that worry you the most				
Safety from bullying, prejudice or discrimination	280	59.5 (54.9–63.8)	191	40.6 (36.2–45.1)
Knowing who to trust	211	44.8 (40.4–49.3)	260	55.2 (50.7–59.6)
Support from parents/caregivers	100	21.2 (17.8–25.2)	371	78.8 (74.8–82.2)
Support from teachers	96	20.4 (17.0–24.3)	375	79.6 (75.7–83.0)
Support from friends	106	22.5 (18.9–26.5)	365	77.5 (73.5–81.1)
Knowing ways to socially transition	95	20.2 (16.8–24.0)	376	79.8 (76.0–83.2)
Support from siblings	66	14.0 (11.2–17.5)	405	86.0 (82.5–88.8)
Something else	33	7.0 (5.0–9.7)	438	93.0 (90.3–95.0)

### Worries and their relationship to social transition extent

Lastly, in a subset of the sample for whom we had complete data on social transition extent for all five contexts (*n* = 208), we examined whether their overall extent of social transition (ranging from a scale of 1–4, see Table 1) differed based on worries about socially transitioning (Supplementary Table S5). The average social transition extent was lower among those with any concerns about socially transitioning compared to those who reported not being concerned about socially transitioning (mean difference = –0.85, 95% CI = –1.27, –0.43), and among those who reported concerns about having support from friends compared to those who did not select this concern (mean difference = –0.8 95% CI = –1.11, –0.49). For all others the differences were smaller.

## Discussion

The comprehensive approach to quantifying social transition taken in this study has demonstrated the complex profile of social transition among young people attending a specialist pediatric gender service. Almost all young people reported either having socially transitioned in some way before their first appointment or intending to do so in the future, expressing desires to change their presentation, name, and/or pronouns. The majority had implemented these changes in at least one context in their lives. However, almost 90% of young people reported at least one concern about doing so. Importantly, by highlighting the complex, heterogenous patterns of social transition across the cohort, our study demonstrated that there is no prescriptive way that young people socially transition.

### Social transition is context-dependent

While some previous research has taken a quantitative approach to examining social transition in multiple contexts, these studies have focused on one aspect of social transition such as change of name (Pollitt et al., 2019), or used contextual data to derive the extent to which young people had socially transitioned overall (Levitan et al., 2019; Sievert et al., 2021). Building on the work of Pollitt et al. (2019), who identified the contexts in which young people were more frequently able to use their chosen name, we found that young people’s *preference* to implement specific social transition practices can differ between contexts, such that young people indicated uncertainty about changing their name and pronouns the most frequently with extended family.

It is essential that young trans people have access to safe environments in which they can socially transition. While we found that a high proportion of young people had socially transitioned in some way in at least one context, when examined across all measures the highest proportions of young people reported having socially transitioned online +/- with friends and at home, and the lowest social transition occurred with extended families and/or schools. It is known that trans young people choose where and how to seek affirmation and express their identities, employing affirmation strategies (e.g. raising awareness and educating others) and survival strategies to protect their safety (e.g. avoiding unsafe places, people, situations) interchangeably (Hill et al., 2025; Pullen Sansfaçon et al., 2021). Taking these factors into account, our finding of relatively less social transition in domestic and education settings (i.e. where young people spend the most time) may suggest that in initial stages of social transition, young people find (or anticipate) online and friends to be affirming environments the most often, extended families and schools the least often, and their homes intermediately. This interpretation does not apply to all young trans people’s circumstances (particularly children under 12 years whose social transition online was not assessed) but may represent a substantial portion of those accessing gender-affirming care. Additionally, our findings raise important questions for future research, such as examining whether less social transition in a specific context is due to a higher degree of anticipated or experienced disaffirmation or discrimination in that setting.

Our findings also show that online spaces are important sites of social transition and affirmation for young trans people. These spaces enable young people some control over their exposure to harmful content and the ability to restrict audiences to improve their safety (Berger et al., 2022), which may be less achievable in non-virtual settings. It is known that young LGBTIQA+ people use online spaces to seek peer connection and knowledge that may be more difficult to obtain in their everyday lives, including connection to queer community, access to queer/trans-related information from online resources or trans peers, to express their identities, or exist in anonymity (Berger et al., 2022; Goffnett & Paceley, 2020; Selkie et al., 2020). Research has additionally shown that avatar creation in video games provides a way for young trans people to explore their identities and transition goals (McKenna et al., 2024). Our findings therefore have implications for policies that seek to restrict young people’s social media access, including in Europe and Australia (Australian Human Rights Commission, 2024).

### Social transition practices and preferences

Compared to name and pronouns, more young people had changed their presentation in every context they wanted to. Therefore, presentation changes may occur initially for the majority of young people’s social transitions, followed by name and pronouns. Longitudinal studies are needed to examine these patterns in the long term.

Change of presentation may additionally have fewer barriers attached, highlighting the relationship between social affirmation—the interpersonal recognition, validation, positive reinforcement, and support of one’s trans identity (King & Gamarel, 2021)—and these different practices of social transition. For instance, changing pronouns and/or name is contingent on the young person’s trans identity being supported by their community, and therefore there may be greater barriers to implementing these practices in some contexts. In contrast—although we did not assess the means by which young people had changed their presentation within each of the examined contexts—presentation changes can be subtle, incremental, and allow young people to explore what feels good to them initially and ongoing. These shifts may occur independent of social affirmation, allowing young people to socially transition to some extent without having to come out to friends or family. In this way, presentation is a form of identity management—ideally giving rise to a passive form of social affirmation where, upon meeting the young person for the first time, people receive visual information about the young person’s gender and respond in ways that are gender-affirming (Doyle, 2022).

However, an important consideration regarding presentation in our clinical experience is that some patients choose to delay social transition until their gender has been medical affirmed and feel their physical appearance better aligns with their gender identity. Understanding differences in social transition preferences and practices in pre-pubertal children, adolescents who intend to medically transition but have not commenced gender-affirming hormones, and those who are receiving hormones, would be important moving forwards.

### Young people’s concerns about social transition may be clinically relevant

We also explored the average extent of social transition in those with and without concerns and found that the extent of average social transition was somewhat different among those concerned about support from friends compared to those who were not, and those with any concerns about socially transitioning compared to those without. Future research is necessary to understand whether those with concerns are transitioning less because of anticipated stigma, less perceived support or other potential reasons, as it has been shown that young trans people who experience stigma personally or vicariously express the need to be vigilant to potential stigma and manage their gender expression to protect themselves from harm (Hill et al., 2025).

Additionally, future research may seek to substantiate how these dual circumstances of being worried about social transition and not having socially transitioned to one’s desired extent materialize in young people’s needs as part of gender-affirming care, as it is likely that the impact of experiencing both these circumstances together require attention. For instance, concerns may reflect the impact of gender minority stressors on young trans people, which are shown to pose significant risks to their safety and wellbeing including greater internalized transphobia, mental health concerns, or fear of identity disclosure (Conn et al., 2023; Dhanani & Totton, 2023; Turban et al., 2021). Additionally, individuals who are not living consistently in their affirmed identities, or who experience gender dysphoria (which may be heightened in those unable to socially transition) are shown to exhibit elevated levels of anxiety, depression or psychological distress (Chodzen et al., 2019; Mezzalira et al., 2022; Vance et al., 2023).

It is also noteworthy that a considerable proportion of young people had socially transitioned in some way while being concerned about social transition. This demonstrates the necessity of social transition in their lives, and their resilience to express and affirm their identities despite social transition being often challenging or fraught. In the present study, young people’s most pertinent concerns were being safe from bullying, prejudice or discrimination (60%) and knowing who to trust (45%) as they navigate social transition. This is in line with previous research showing that individuals who socially transition in childhood or adolescence more frequently report gender-related harassment experiences than those who socially transition in adulthood (Turban et al., 2021), and young people are evidently aware that other people may expose them to harm. That relatively lower proportions reported concern about being supported by teachers, parents/caregivers or friends (20%–22%) makes it is possible that for the majority, concerns about discrimination related prim′arily to their safety in public or at school more broadly, rather than those to whom they are closest.

### Quantifying social transition

In the present political climate, where misinformation about social transition is widespread (e.g. concerns that social transition causes the persistence of trans identity or is not beneficial to young trans people’s mental health (Cass, 2024)), it has been argued that measures of social transition should be designed to examine young trans people’s genuine lived experiences, and convey these experiences first-hand (Horton, 2024). This is to ensure that future research involving measures of social transition can avoid the pitfalls of past studies, including the reliance on clinician-reported data or erasure of important contextual information that is likely to mediate or confound the causal relationship between social transition and wellbeing (Horton, 2024).

The present study has provided a comprehensive profile of social transition by capturing young people’s own reports of their social transition contexts, preferences, practices, extent and concerns. Portraying a clear picture of where changes are—and are not—taking place can enable greater understanding of youth social transition as a complex process rather than a binary phenomenon. Coupled with knowledge of young people’s worries about socially transitioning, this comprehensive profile can show where young people are encountering resistance or barriers to social transition, and where they prefer to transition initially (Horton, 2024). When taking both preference and context into consideration, it is evident that different practices of social transition do not involve binary changes but are processes in and of themselves. If we were to treat change in name, for example, as a binary construct without ascertaining preference or where this change had occurred, we would observe that 73.9% of the sample had changed their name. However, only 45% of those classified in this way as having changed their name did so in every context, and 20% of those classified as having not changed their name had no intention to do so. These are important nuances that are likely to have been missed in previous research and to have affected the reliability of past results, and future research must bear this in mind.

As shown in Figure 4, some young people had implemented all the examined practices across all contexts, while others had implemented none, and there were also many young people who exhibited heterogeneous social transition preferences and practices across contexts. Moving forwards, to reduce the risk of reinforcing simplistic, binary notions of transition that trans people may internalize (Garrison, 2018), it will be important for researchers and clinicians to avoid quantifying ‘full’ and ‘partial’ social transition based on a prescriptive set of practices that pre-suppose ‘full social transition’ is a universal end-goal or assume that these preferences won’t change over time. Our study has shown that quantifying social transition can be achieved in a way that is both nuanced *and* in accordance with young people’s own goals, whatever they may be.

#### Strengths and limitations

Nonbinary and non-dysphoric trans youths’ experiences have been overlooked in some of the available literature on social transition (Durwood et al., 2017; Morandini et al., 2023). We included young people irrespective of gender dysphoria diagnosis or gender identity—an additional strength of this study which better reflects the current profile of young people accessing gender-affirming care.

We are nonetheless aware that we have not addressed all pitfalls of research on young people’s social transition (Horton, 2024) and encourage other researchers to improve upon our work. Firstly, we recognize these data were collected as part of clinical care, and that socially transitioning in certain ways was historically a requirement to access gender-affirming medical care. The legacy of this narrow eligibility persists today in both the practices of some providers and understanding among young people and their caregivers that a lack of social transition may undermine one’s access to care (Chong et al., 2021; Horton, 2022; Kearns et al., 2021). It is therefore possible that young people felt it necessary to overstate their social transition.

Secondly, most young people were of non-racialized backgrounds and lived in socioeconomically advantaged neighborhoods, and all were accessing specialist gender-affirming care which typically relies on having at least one supportive parent. As Pullen Sansfaçon et al. (2021) explicate through the narratives of trans youth, those who are white (particularly those who are also cis-passing) receive greater opportunity than those who are racialized to deploy affirmation strategies, survival strategies or both in tandem by virtue of these intersecting privileges. In this way, further quantitative research is needed to better capture how young people experiencing multiple forms of marginalization or disadvantage negotiate their social transitions.

Thirdly, although the measures used in the present study attempt to quantify social transition comprehensively, the measures were not co-designed, and examining their performance and validation was beyond scope. We did not capture all practices of social transition or gender affirmation and may have invertedly provided a framework through which young people assessed how much they’d socially transitioned based on the examined practices alone. We also did not specifically ask about potentially fluid, non-linear social transition experiences, although these may be captured if the survey was administered longitudinally. We also used proximal measures to understand preferences for name, presentation and pronoun changes. Additionally, because of the structure of the questions (see Supplementary Table S1), we caution that having transitioned ‘As much as I can’ may refer to young people having made all their desired changes, or only some due to barriers to doing so. Similarly, ‘One day’ responses (e.g. to the question ‘have you changed your pronouns at home?’) may not demonstrate barriers to making these changes but that young people are simply not ready. Based on the findings of past research depicting barriers to social transition in child and youth cohorts (Belmont et al., 2024; Horton, 2023), we cautiously believe similar circumstances to be inclusive of most participants’ experiences, although the results should be interpreted with these limitations in mind.

Fourthly, it will be important for future measures of social transition to capture how diverse groups of young trans people geographically, economically and fluidly negotiate social transition. While some of these factors have been captured qualitatively (de Castro Peraza et al., 2023; Horton, 2023; Pullen Sansfaçon et al., 2020, 2021, 2023), co-designed quantitative measures are necessary to better reflect the experiences of young trans people when attempting to understand processes of social transition or its psychosocial outcomes.

Finally, it is important to note that our current analysis was not designed to determine the causal effects of young people’s concerns on their social transition. Instead, our study’s aim in this regard was descriptive, with the goal being to compare the extent of social transition among those with and without concerns. While this comparison did allow us to determine which group was more or less likely to transition, the differences that we observed cannot solely be attributed to having concerns given the likely presence of confounding factors that are different between the two groups (e.g. degree of support). Looking ahead, future research that adjusts for confounders and determines whether concerns (and other relevant factors) have a causal influence on social transition would be valuable.

### Clinical recommendations

Aligned with clinical guidelines (Coleman et al., 2022; Telfer), provision of safe and appropriate support to a young trans person should involve discussion of the young person’s social transition goals, ascertaining whether social transition is desired and what practices the young person intends to address where and when. While 20% of young people reported concern about not knowing ways to socially transition, practical strategies to facilitate access to affirming environments in which they can safely explore and attain these goals at an appropriate pace (e.g. in collaboration with schools and families, and/or in controlled online environments if the young person is of an appropriate age; particularly if social transition is not possible in the former settings) may be a bigger priority for the other 80% of patients. If young people wish to socially transition at home, school and with extended families, specific support may be required to facilitate these changes.

## Conclusions

This study has shown that a young person’s social transition goals are nuanced and may be contextually situated. Neglecting these details in quantitative assessment can obscure the ways young people navigate their transition across different contexts that may confer varying levels of safety, affirmation or barriers to transition. Online spaces and friendships are important sites of social transition to young trans people, while social transition may occur to a lesser extent with extended families, at schools and in young people’s homes, at least initially. It is also evident that many young people socially transition despite having concerns about undergoing this process, and these circumstances should be given attention in gender-affirming care.

Looking ahead, we encourage the use of nuanced, in-depth quantitative measures which avoid reinforcing binary, transnormative notions of social transition, since this should improve understanding of young trans people’s social transition processes and ensure that future policy recommendations and clinical guidelines are informed by the genuine experiences of young trans people. While the present study contributes toward this goal, there is also a need for measures of social transition that are co-designed with young people and better capture their aspirations, practices and experiences (including their social and political context). Finally, understanding youth social transition beyond the experiences of those accessing gender-affirming care, and including young people who hold multiple marginalized identities, will help create safer environments in which to socially transition for all young trans people.

## Supplementary Material

Supplement.docx

## Data Availability

We will consider making the data that support the findings of this study available, including de-identified participant data and data dictionaries, upon reasonable request. Researchers requesting these data will need to submit a methodologically sound proposal. Proposals should be directed to corresponding author Ken Pang (ken.pang@mcri.edu.au) for consideration. Data will only be shared after the proposal has been approved and once any necessary ethics approvals have been obtained. Once the data are shared, they can only be used for the purposes outlined in the approved proposal. To gain access to data, requestors will need to sign a data access agreement.
