# Supplementary material for: Social transition in young trans people accessing gender-affirming care
Source: Int J Transgend Health. 2025 Jun 20;27(5):2332–50. doi: 10.1080/26895269.2025.2516678 (PMC13618105; doi:10.1080/26895269.2025.2516678)
Supplement: Supplementary file 1 [file WIJT_A_2516678_SM6845.docx]

### Supplementary Files

Table S1. Social transition questionnaire

*About social transition*

Social transition is where someone changes the way they present themselves to more closely match their gender identity. For example, someone who socially transitions might change their name or nickname, their hair or clothes, the activities they do, the toilets they use at school, and other things. Some people don't socially transition at all; others might transition only at home, at school/TAFE/university, at many places, or everywhere. They might transition in some ways or in many ways. They might transition in steps or all at once.

**Have you socially transitioned in any way?***

[ ] Yes

[ ] No

**Do you think you might want to socially transition, in any way, one day?***

[ ] Yes

[ ] No

*Extent of social transition*

**Overall, I have socially transitioned...****

|  | In no ways | In some ways | In many ways | As much as I can | Not applicable |
| --- | --- | --- | --- | --- | --- |
|  |  |  |  |  |  |
| At home | [ ] | [ ] | [ ] | [ ] | [ ] |
| At school/other place of study | [ ] | [ ] | [ ] | [ ] | [ ] |
| Online*** | [ ] | [ ] | [ ] | [ ] | [ ] |
| With Friends | [ ] | [ ] | [ ] | [ ] | [ ] |
| With Extended family (e.g., grandparents, cousins, etc.) | [ ] | [ ] | [ ] | [ ] | [ ] |

*Social transition practices*

Next, we ask about things that some people change when they socially transition and some of the places these changes might happen.

**About my 'look' (clothes and any accessories, hairstyle, makeup, etc.)**

| To suit my gender identity… | I have changed my look | I don’t want to change my look | One day, I will change my look | I don’t know | Not applicable |
| --- | --- | --- | --- | --- | --- |
|  |  |  |  |  |  |
| At home | [ ] | [ ] | [ ] | [ ] | [ ] |
| At school/other place of study | [ ] | [ ] | [ ] | [ ] | [ ] |
| Online | [ ] | [ ] | [ ] | [ ] | [ ] |
| With Friends** | [ ] | [ ] | [ ] | [ ] | [ ] |
| With Extended family (e.g., grandparents, cousins, etc.)** | [ ] | [ ] | [ ] | [ ] | [ ] |

**About my name**

| To suit my gender identity… | I have changed my name | I don’t want to change my name | One day, I will change my name | I don’t know | Not applicable |
| --- | --- | --- | --- | --- | --- |
|  |  |  |  |  |  |
| At home | [ ] | [ ] | [ ] | [ ] | [ ] |
| At school/other place of study | [ ] | [ ] | [ ] | [ ] | [ ] |
| Online | [ ] | [ ] | [ ] | [ ] | [ ] |
| With Friends | [ ] | [ ] | [ ] | [ ] | [ ] |
| With Extended family (e.g., grandparents, cousins, etc.) | [ ] | [ ] | [ ] | [ ] | [ ] |

**About my pronouns (he, she, they, etc.)**

| To suit my gender identity… | I have changed my pronouns | I don’t want to change my pronouns | One day, I will change my pronouns | I don’t know | Not applicable |
| --- | --- | --- | --- | --- | --- |
|  |  |  |  |  |  |
| At home | [ ] | [ ] | [ ] | [ ] | [ ] |
| At school/other place of study | [ ] | [ ] | [ ] | [ ] | [ ] |
| Online | [ ] | [ ] | [ ] | [ ] | [ ] |
| With Friends** | [ ] | [ ] | [ ] | [ ] | [ ] |
| With Extended family (e.g., grandparents, cousins, etc.)** | [ ] | [ ] | [ ] | [ ] | [ ] |

*Worries about socially transitioning*

**Thinking about socially transitioning, please select up to three of the options that most worry you right now.**

| I’m worried about… | [ ] | Having support from my parents or caregivers |
| --- | --- | --- |
|  | [ ] | Having support from my siblings |
|  | [ ] | Having support from my school teachers/lecturers/tutors |
|  | [ ] | Having support from my friends |
|  | [ ] | Knowing which people I can trust |
|  | [ ] | Knowing ways I could socially transition |
|  | [ ] | Being safe from bullying, prejudice, discrimination, etc. |
|  | [ ] | I don’t know |
|  | [ ] | I’m not worried about socially transitioning |
|  | [ ] | I prefer not to answer |
|  | [ ] | Other: |

*Questions used to determine the analytic sample.

**Questions added into the questionnaire on 2^nd^ August 2018.

Table S2. Demographic comparison of young people who submitted surveys before 2 August 2018 or on/after this date, when additional questions were implemented

|  | Before 2 Aug 2018 | | 2 Aug 2018 or later | |
| --- | --- | --- | --- | --- |
|  | N | % (95% CI) | N | % (95% CI) |
| Gender |  |  |  |  |
| Boy | 134 | 54.9 (48.6-61.1) | 123 | 52.1 (45.7-58.5) |
| Girl | 44 | 18 (13.7-23.4) | 43 | 18.2 (13.8-23.7) |
| Nonbinary | 33 | 13.5 (9.8-18.4) | 45 | 19.1 (14.5-24.6) |
| Unsure | 31 | 12.7 (9.1-17.5) | 25 | 10.6 (7.2-15.2) |
| Prefer not to answer | 2 | 0.8 (0.2-3.2) | 0 | 0.0 |
| Missing | 2 |  | 0 |  |
| Age |  |  |  |  |
| 8-11 years | 35 | 14.2 (10.4-19.2) | 40 | 16.9 (12.7-22.3) |
| 12-17 years | 211 | 85.8 (80.8-89.6) | 196 | 83.1 (77.7-87.3) |
| Mean (SD) | 246 | 14.6 (2.2) | 236 | 14.4 (2.2) |
| Missing | 0 |  | 0 |  |
| Country of origin |  |  |  |  |
| Australia | 230 | 94.3 (90.5-96.6) | 215 | 91.9 (87.6-94.8) |
| Other | 14 | 5.7 (3.4-9.5) | 19 | 8.1 (5.2-12.4) |
| Missing | 2 |  | 2 |  |
| Main language spoken at home |  |  |  |  |
| English | 235 | 97.9 (95.1-99.1) | 230 | 100.0 |
| Other | 5 | 2.1 (0.9-4.9) | 0 | 0.0 |
| Missing | 6 |  | 6 |  |
| Aboriginal and/or Torres Strait Islander |  |  |  |  |
| Yes | 2 | 0.9 (0.2-3.5) | 226 | 4.2 (2.3-7.7) |
| No | 223 | 99.1 (96.5-99.8) | 10 | 95.8 (92.3-97.7) |
| Missing | 21 |  | 0 |  |
| Attends school |  |  |  |  |
| Yes | 226 | 94.6 (90.8-96.8) | 216 | 93.9 (90.0-96.4) |
| No | 13 | 5.4 (3.2-9.2) | 14 | 6.1 (3.6-10.0) |
| Missing | 7 |  | 6 |  |
| School type |  |  |  |  |
| Government (public) | 140 | 65.1 (58.5-71.2) | 153 | 73.6 (67.1-79.1) |
| Independent (private) | 56 | 26 (20.6-32.4) | 40 | 19.2 (14.4-25.2) |
| Other | 19 | 8.8 (5.7-13.5) | 15 | 7.2 (4.4-11.6) |
| Missing | 18 |  | 14 |  |
| Socioeconomic status (IRSAD quintile) |  |  |  |  |
| 1 | 28 | 11.4 (8.0-16.0) | 17 | 7.2 (4.5-11.4) |
| 2 | 31 | 12.6 (9.0-17.4) | 30 | 12.8 (9.1-17.7) |
| 3 | 43 | 17.5 (13.2-22.8) | 47 | 20 (15.3-25.6) |
| 4 | 69 | 28.0 (22.8-34.0) | 68 | 28.9 (23.5-35.1) |
| 5 | 75 | 30.5 (25.0-36.6) | 73 | 31.1 (25.4-37.3) |
| Missing | 0 |  | 1 |  |
| Residential location |  |  |  |  |
| Metropolitan | 164 | 66.9 (60.8-72.6) | 166 | 70.6 (64.5-76.1) |
| Regional center | 37 | 15.1 (11.1-20.2) | 23 | 9.8 (6.6-14.3) |
| Large rural town | 3 | 1.2 (0.4-3.7) | 6 | 2.6 (1.1-5.6) |
| Med rural town | 4 | 1.6 (0.6-4.3) | 12 | 5.1 (2.9-8.8) |
| Small rural town | 37 | 15.1 (11.1-20.2) | 28 | 11.9 (8.3-16.7) |
| Missing | 1 |  | 1 |  |

**Table S3. Extent of social transition at home, school, online, with friends and with extended family.**

| **Socially transitioned:** | **In no ways** | | **In some ways** | | **In many ways** | | **As much as I can** | | **Any** | |
| --- | --- | --- | --- | --- | --- | --- | --- | --- | --- | --- |
|  | **n** | **% (95% CI)** | **n** | **% (95% CI)** | **n** | **% (95% CI)** | **n** | **% (95% CI)** | **n** | **% (95% CI)** |
| Home (n=234) | 34 | 14.5 (10.6-19.7) | 57 | 24.4 (19.3-30.3) | 34 | 14.5 (10.6-19.7) | 109 | 46.6 (40.2-53.0) | 200 | 85.5 (80.3-89.4) |
| School (n=226) | 39 | 17.3 (12.8-22.8) | 57 | 25.2 (20.0-31.3) | 32 | 14.2 (10.2-19.4) | 98 | 43.4 (37.0-49.9) | 187 | 82.7 (77.2-87.2) |
| Online (n=186) | 25 | 13.4 (9.2-19.2) | 22 | 11.8 (7.9-17.4) | 24 | 12.9 (8.8-18.6) | 115 | 61.8 (54.6-68.6) | 161 | 86.6 (80.8-90.8) |
| Friends (n=233) | 31 | 13.3 (9.5-18.3) | 43 | 18.5 (14.0-24.0) | 34 | 14.6 (10.6-19.8) | 125 | 53.6 (47.2-60.0) | 202 | 86.7 (81.7-90.5) |
| Extended family (n=224) | 61 | 27.2 (21.8-33.5) | 75 | 33.48 (27.6-40.0) | 24 | 10.7 (7.3-15.5) | 64 | 28.6 (23.0-34.9) | 163 | 72.8 (66.5-78.2) |

Table S4. Preference and implementation of social transition practices at home, school, online, with friends and with extended family

| **Change:** | **Presentation** | | **Name** | | **Pronouns** | |
| --- | --- | --- | --- | --- | --- | --- |
|  | n | % (95% CI) | n | % (95% CI) | n | % (95% CI) |
| Home |  |  |  |  |  |  |
| Preference: | 476 |  | 469 |  | 473 |  |
| Yes | 447 | 93.9 (91.4-95.7) | 400 | 85.3 (81.8-88.2) | 426 | 90.1 (87.0-92.5) |
| No | 11 | 2.3 (1.3-4.1) | 26 | 5.5 (3.8-8.0) | 5 | 1.1 (0.4-2.5) |
| Don’t know | 18 | 3.8 (2.4-5.9) | 43 | 9.2 (6.9-12.1) | 42 | 8.9 (6.6-11.8) |
| Done: |  |  |  |  |  |  |
| Yes | 370 | 82.8 (79.0-86.0) | 247 | 61.8 (56.9-66.4) | 274 | 64.3 (59.6-68.7) |
| No, but will in the future | 77 | 17.2 (14.0-21.0) | 153 | 38.2 (33.6-43.1) | 152 | 35.7 (31.3-40.4) |
| School |  |  |  |  |  |  |
| Preference: | 458 |  | 454 |  | 460 |  |
| Yes | 429 | 93.7 (91.0-95.6) | 385 | 84.8 (81.2-87.8) | 413 | 89.8 (86.7-92.2) |
| No | 12 | 2.6 (1.5-4.6) | 28 | 6.2 (4.3-8.8) | 8 | 1.7 (0.9-3.4) |
| Don’t know | 17 | 3.7 (2.3-5.9) | 41 | 9.0 (6.7-12.0) | 39 | 8.5 (6.3-11.4) |
| Done: |  |  |  |  |  |  |
| Yes | 335 | 78.1 (73.9-81.8) | 225 | 58.4 (53.4-63.3) | 239 | 57.9 (53.0-62.6) |
| No, but will in the future | 94 | 21.9 (18.2-26.1) | 160 | 41.6 (36.7-46.6) | 174 | 42.1 (37.4-47.0) |
| Online |  |  |  |  |  |  |
| Preference: | 344 |  | 386 |  | 388 |  |
| Yes | 308 | 89.5 (85.8-92.4) | 354 | 91.7 (88.5-94.1) | 370 | 95.4 (92.7-97.1) |
| No | 7 | 2.0 (1.0-4.2) | 14 | 3.6 (2.2-6.0) | 1 | 0.3 (0.0-1.8) |
| Don’t know | 29 | 8.4 (5.9-11.9) | 18 | 4.7 (3.0-7.3) | 17 | 4.4 (2.7-6.9) |
| Done: |  |  |  |  |  |  |
| Yes | 271 | 88.0 (83.8-91.2) | 313 | 88.4 (84.6-91.4) | 323 | 87.3 (83.5-90.3) |
| No, but will in the future | 37 | 12.0 (8.8-16.2) | 41 | 11.6 (8.6-15.4) | 47 | 12.7 (9.7-16.5) |
| Friends |  |  |  |  |  |  |
| Preference: | 233 |  | 228 |  | 232 |  |
| Yes | 225 | 96.6 (93.3-98.3) | 201 | 88.2 (83.3-91.8) | 215 | 92.7 (88.5-95.4) |
| No | 2 | 0.9 (0.2-3.4) | 16 | 7.0 (4.3-11.2) | 1 | 0.4 (0.1-3.0) |
| Don’t know | 6 | 2.6 (1.2-5.6) | 11 | 4.8 (2.7-8.5) | 16 | 6.9 (4.3-11.0) |
| Done: |  |  |  |  |  |  |
| Yes | 185 | 82.2 (76.6-86.7) | 157 | 78.1 (71.8-83.3) | 173 | 80.5 (74.6-85.3) |
| No, but will in the future | 40 | 17.8 (13.3-23.4) | 44 | 21.9 (16.7-28.2) | 42 | 19.5 (14.7-25.4) |
| Extended family |  |  |  |  |  |  |
| Preference: | 222 |  | 220 |  | 222 |  |
| Yes | 203 | 91.4 (86.9-94.5) | 163 | 74.1 (67.9-79.5) | 173 | 77.9 (72.0-82.9) |
| No | 4 | 1.8 (0.7-4.7) | 19 | 8.6 (5.6-13.2) | 5 | 2.3 (0.9-5.3) |
| Don’t know | 15 | 6.8 (4.1-10.9) | 38 | 17.3 (12.8-22.9) | 44 | 19.8 (15.1-25.6) |
| Done: |  |  |  |  |  |  |
| Yes | 160 | 78.8 (72.6-83.9) | 74 | 45.4 (37.9-53.1) | 73 | 42.2 (35.0-49.7) |
| No, but will in the future | 43 | 21.2 (16.1-27.4) | 89 | 54.6 (46.9-62.1) | 100 | 57.8 (50.3-65.0) |

Preference defined by proxy of having implemented the practice (‘Done’) or intending to in the future (‘One day’).

Table S5. Difference in extent of social transition between young people who reported worries or did not report worries in the ‘extent scale’ subsample (n=208)

| Reported worrying about | Yes | | | No | | | Difference, overall extent of social transition |
| --- | --- | --- | --- | --- | --- | --- | --- |
|  | n | % (95% CI) | Overall extent of social transition, mean (95% CI) | n | % (95% CI) | Overall extent of social transition, mean (95% CI) | b (95% CI) |
| Safety from bullying, prejudice or discrimination | 123 | 60.3 (53.4-66.8) | 2.9 (2.7-3.0) | 81 | 39.7 (33.2-46.6) | 2.8 (2.6-3.1) | 0.1 (-0.2-0.3) |
| Knowing who to trust | 96 | 47.1 (40.3-54.0) | 2.9 (2.7-3.1) | 108 | 52.9 (46.0-59.7) | 2.9 (2.7-3.0) | 0.0 (-0.2-0.3) |
| Support from parents/caregivers | 38 | 18.6 (13.8-24.6) | 2.7 (2.4-2.9) | 166 | 81.4 (75.4-86.2) | 2.9 (2.8-3.1) | -0.3 (-0.6-0.1) |
| Support from friends | 43 | 21.1 (16.0-27.3) | 2.2 (2.0-2.5) | 161 | 78.9 (72.7-84.0) | 3.0 (2.9-3.2) | -0.8 (-1.1--0.5) |
| Knowing ways to socially transition | 42 | 20.6 (15.6-26.7) | 2.7 (2.5-3.0) | 162 | 79.4 (73.3-84.4) | 2.9 (2.7-3.0) | -0.1 (-0.5-0.2) |
| Support from teachers | 41 | 20.1 (15.1-26.2) | 2.8 (2.5-3.1) | 163 | 79.9 (73.8-84.9) | 2.9 (2.7-3.0) | -0.1 (-0.4-0.3) |
| Support from siblings | 26 | 12.7 (8.8-18.1) | 2.7 (2.4-3.1) | 178 | 87.3 (81.9-91.2) | 2.9 (2.7-3.0) | -0.1 (-0.5-0.3) |
| Something else | 13 | 6.4 (3.7-10.7) | 3.3 (2.8-3.7) | 191 | 93.6 (89.3-96.3) | 2.8 (2.7-3.0) | 0.4 (-0.1-1.0) |
| Any of the above | 164 | 88.2 (82.6-92.1) | 2.8 (2.7-3.0) | 22 | 11.8 (7.9-17.4) | 3.7 (3.4-3.9) | -0.8 (-1.3--0.4) |

Overall extent of social transition scores range from 1 to 4, with 1 denoting no social transition (i.e., transitioned ‘in no ways’ in every context) and 4 denoting ‘as much as possible’ extent of social transition in every context.

For analyses involving the individual ‘worry’ items, comparisons are reported as the mean difference (95% CI) in extent of social transition in those who indicated [item] was one of the three options that worried them most about socially transitioning, compared to those who did not select this item. The analysis involving the ‘any worries’ variable is reported as the mean difference in extent of social transition in young people who reported any worries about social transitioning compared to those who reported not being worried about socially transitioning.
